# Schisantherin B Preserves Cochlear Function and Hair Cell Integrity in Aging by Restoring Mitochondrial Homeostasis via eEF1A2–Beclin‐1–Mediated Mitophagy

**DOI:** 10.1002/cns.71267

**Published:** 2026-11-13

**Authors:** Gongrui Tang, Ziwen Gao, Tianyu Ma, Mengting Liu, Jiangping Xie, Ping yang, Yilai Shu, Tianhong Zhang

**Affiliations:** ^1^ Department of Otorhinolaryngology Head and Neck Surgery The First Hospital Affiliated to Harbin Medical University Harbin Heilongjiang China; ^2^ ENT Institute and Department of Otorhinolaryngology, Eye & ENT Hospital Fudan University Shanghai China; ^3^ Shanghai Key Laboratory of Gene Editing and Cell Therapy for Rare Diseases Fudan University Shanghai China; ^4^ State Key Laboratory of Brain Function and Disorders and MOE Frontiers Center for Brain Science Fudan University Shanghai China; ^5^ NHC Key Laboratory of Hearing Medicine Shanghai China; ^6^ Institutes of Biomedical Sciences Fudan University Shanghai China; ^7^ Institute of Medical Genetics and Genomics Fudan University Shanghai China; ^8^ Shanghai Academy of Natural Sciences (SANS) Shanghai China; ^9^ FuRong Laboratory Changsha Hunan China

**Keywords:** age‐related hearing loss, Chinese medicine, mitophagy, Schisantherin B

## Abstract

**Background:**

Age‐related hearing loss (ARHL) represents one of the prevalent degenerative conditions in the elderly worldwide, characterized by progressive auditory dysfunction that impairs quality of life. Currently, no approved pharmacotherapies are available to prevent.

**Methods:**

In vitro protective effects of STB were assessed by cell viability and Senescence‐associated β‐galactosidase (SA‐β‐gal staining) in D‐galactose‐induced senescent mouse cochlear explants and HEI‐OC1 cells. In vivo auditory function and cochlear injury were examined by hearing threshold detection and histological analysis of hair cells, ribbon synapses, and spiral ganglion neurons in SAMP8 and C57BL/6J mice. RNA sequencing was used to identify STB‐mediated transcriptional changes. Immunofluorescence and Western blotting were performed to investigate the molecular mechanisms.

**Results:**

STB pretreatment reduced cell loss and senescence in vitro. In vivo, STB attenuated low‐to‐mid frequency hearing threshold shifts and cochlear degeneration. Mechanistically, STB upregulated eEF1A2, which promoted Beclin‐1‐mediated mitophagy, reduced ROS accumulation, and suppressed caspase‐3‐dependent apoptosis. RNA‐seq confirmed that STB modulated Beclin‐1 expression via eEF1A2.

**Conclusion:**

These data confirm the significant protective effect of STB against ARHL, highlighting its potential as a natural candidate for ARHL intervention and verify that it exerts its effects by regulating cellular autophagy via the eEF1A2‐Beclin 1 pathway.

## Graphical abstract

STB delays age‐related hearing loss in vivo (SAMP8 mice, C57BL/6J mice) and in vitro (HEI‐OC1 cells, cochlear explants). RNAseq reveals that STB acts through eEF1A2‐Beclin‐1‐mediated mitophagy to reduce apoptosis in auditory cells.

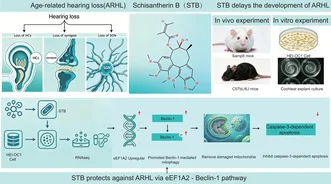

## 1 Introduction

Age‐related hearing loss (ARHL), also known as presbycusis, is emerging as one of the most common chronic conditions in the elderly in light of the global trend toward an aging population [1]. It is characterized by bilateral and progressive damage to the inner ear, particularly affecting cochlear hair cells (HCs), synaptic connections, and spiral ganglion neurons (SGNs) [2, 3]. Accumulating evidence increasingly associates ARHL with cognitive decline and an elevated risk of dementia, posing a substantial public health challenge [4]. Clinical management of ARHL remains limited, with interventions primarily relying on assistive devices such as hearing aids and cochlear implants [5]. No approved pharmacological interventions currently exist to alleviate its burden on individuals and society.

Schisantherin B (STB), a bioactive lignan from Schisandra chinensis—a traditional Chinese herb with “kidney‐tonifying and heart‐calming” properties—has demonstrated diverse biological activities in recent decades, notably neuroprotection [6, 7]. STB has been shown to alleviate Aβ₁₋₄_2_‐induced neurotoxicity by restoring GLT‐1 expression in AD mouse models, improving cognitive dysfunction and protecting against CNS degeneration, and to ameliorate pathological features, depressive‐like behaviors, and cognitive deficits in age‐specific depressive mouse models, collectively supporting its regulatory role in neurological and emotion‐related disorders [8, 9]. Given STB's extensive research foundation in neuroprotection and the shared pathological mechanisms between ARHL and neurodegenerative diseases (e.g., cellular senescence, apoptosis, oxidative stress) [6], STB is speculated to exert potential protective effects on ARHL, providing an important theoretical basis for the present study.

ARHL is a complex disease involving multiple underlying biological mechanisms, among which autophagic dysfunction is considered an important contributor to its onset and progression [7]. Accumulating evidence has demonstrated that the normal activation of autophagy is crucial for maintaining cochlear cellular homeostasis and delaying ARHL progression [10]. Liu et al. [11] demonstrated that rapamycin improves ARHL symptoms in C57BL/6J mice by enhancing SGN autophagy. Lee, Y. Y [12]. showed that abnormal cholesterol metabolism and lysosomal dysfunction can induce ARHL by inhibiting the mTORC1‐TFEB‐dependent autophagic pathway. There is a paucity of specific interventional drugs capable of delaying or inhibiting disease progression at the level of pathological mechanisms [13]. Consequently, the investigation of potential active compounds for combating ARHL and elucidating their underlying mechanisms has become a focal point of research in auditory medicine.

This study established in vitro aging models using cochlear explants and HEI‐OC1 cells and demonstrated the protective effects of STB against D‐gal‐induced senescence. Subsequently, in vivo validation was performed using Senescence‐Accelerated Mouse Prone 8/Senescence‐Accelerated Mouse Resistant 1 (SAMP8/SAMR1) and C57BL/6J mice [13, 14] and the combined use of these two models enabled evaluation of STB's protective effect on ARHL. RNA‐seq further identified eEF1A2 as a potential STB‐responsive molecule, while subsequent experiments suggested that the protective effects of STB were associated with the regulation of autophagy and Beclin‐1‐related signaling [15]. These findings provide experimental evidence supporting the potential of STB as a candidate for ARHL intervention and offer insights into its underlying molecular mechanisms.

## 2 Methods

### 2.1 Organotypic Culture of the Cochlea From Neonatal Mice

All experimental procedures involving animals in this study were conducted in accordance with animal research ethics and policies, and received approval from the Institutional Animal Care and Use Committees of both Fudan University and the First Affiliated Hospital of Harbin Medical University. (No: 202410065S; IACUC‐2023092). Postnatal Day 2 (P2) ICR mice were humanely euthanized, and cochlear tissues were aseptically dissected under a stereomicroscope. Adjacent connective and non‐sensory tissues were meticulously removed. Isolated cochlear explants were transferred to Cell‐Tak (BD Biosciences, San Jose, CA, USA)‐precoated culture dishes and maintained in DMEM/F12 medium supplemented with N2, B27 (Thermo Fisher Scientific, Waltham, MA, USA), and ampicillin. Prior to subsequent experimental treatments, the explant cultures were incubated overnight at 37°C in a humidified 5% CO_2_ atmosphere.

### 2.2 HEI‐OC1 Cell Culture

HEI‐OC1 cells were propagated in high‐glucose DMEM supplemented with 5% fetal bovine serum (FBS, Thermo Fisher Scientific, Waltham, MA, USA). The cultures were incubated at 33°C in a humidified atmosphere with 5% CO_2_.

### 2.3 Drug Treatments

STB was procured from Shanghai Kingmorn Biotechnology Co. Ltd. (Shanghai, China) and subsequently dissolved in dimethyl sulfoxide (DMSO, Sigma‐Aldrich, St. Louis, MO, USA) to create a 4 mM stock solution. For experimental applications, this stock solution was diluted to achieve the desired final concentrations of 10, 50, 100, and 200 μM. D‐galactose (D‐gal) was obtained from Sigma‐Aldrich (USA) and dissolved in double‐distilled water (ddH_2_O) to prepare solutions at concentrations of 20, 40, and 60 mg/mL.

### 2.4 Animal Treatments

Male wild‐type SAMP8, SAMR1, and C57BL/6J mice were used in this study. For the SAMP8 aging model, one‐month‐old SAMP8 mice were randomly assigned to the untreated SAMP8 group (*n* = 18) or the STB‐treated SAMP8 group (*n* = 18), while age‐matched SAMR1 mice (*n* = 8) served as controls. Mice in the STB‐treated group received 60 mg/kg STB, as determined by a preliminary dose–response study (Figure S1), via intragastric administration every other day, whereas untreated and control mice received an equivalent volume of sterile normal saline. Auditory Brainstem Response (ABR) measurements were performed at baseline and at 3 and 6 months after the initiation of STB administration.

For the naturally aged model, 12‐month‐old male wild‐type C57BL/6J mice were randomly assigned to the untreated aging group (*n* = 12) or the STB‐treated aging group (*n* = 12). STB administration was performed using the same dose and regimen described above, while the untreated aging group received an equivalent volume of sterile normal saline. Four‐month‐old C57BL/6J mice were included as young controls for comparison with age‐related changes.

For both animal models, mice were randomly allocated to the experimental groups, and investigators responsible for ABR measurements, histological assessments, and data analysis were blinded to group allocation. The dosage of STB and the experimental protocol were based on established studies evaluating protective agents in murine models.

### 2.5 Cell Viability Assay

Cell viability was assessed using the CCK‐8 assay kit (Sigma‐Aldrich, St. Louis, MO, USA). Briefly, HEI‐OC1 cells from each experimental cohort were rinsed with pre‐warmed phosphate‐buffered saline (PBS), followed by incubation with CCK‐8 solution for 2 h. Using a microplate reader (Bio‐Rad Laboratories, Hercules, CA, USA), the absorbance was read at 450 nm. Blank control wells (without cells) and negative control wells (untreated controls) were processed in parallel under identical conditions. Each experimental group included three technical replicates.

### 2.6 Annexin V/PI Flow Cytometry Analysis

For quantitative apoptosis analysis, cells were stained using an Annexin V‐FITC kit (BD Biosciences Pharmingen, San Diego, CA, USA) per the manufacturer's protocol. Briefly, cells were washed with PBS and resuspended in 1 × binding buffer, followed by incubation with 5 μL of Annexin V‐FITC and 5 μL of propidium iodide (PI) for 15 min at room temperature in the dark. A FACS Calibur flow cytometer (BD Biosciences) was used to acquire 10,000–20,000 events per sample, and data were analyzed with FlowJo 7.6 software. The experiment was performed in triplicate with three technical replicates each.

### 2.7 Detection of Caspase‐3/7 Activity

HEI‐OC1 cells were washed with serum‐free DMEM and then incubated with 5 μmol/L Caspase‐3/7 green detection reagent (Thermo Fisher Scientific, USA) at 37°C for 30 min. Caspase‐3/7 activity was subsequently assessed by fluorescence microscopy and flow cytometry.

### 2.8 TUNEL Assay

Apoptotic cells were detected using an in situ cell death detection kit (Roche, Indianapolis, IN, USA) according to the manufacturer's protocol. Following DAPI and primary antibody labeling, specimens were visualized and imaged under a Leica TCS SP8 confocal laser scanning microscope (Leica Microsystems, Wetzlar, Germany).

### 2.9 ROS And MMP Assays

Cellular ROS and MMP were detected using CellROX Green reagent (Thermo Fisher Scientific, USA) and Rhodamine 123, respectively. For MMP measurement, cells were incubated with 1 μmol/L Rhodamine 123 for 30 min at 37°C in darkness. Fluorescence was analyzed by confocal microscopy (Leica SP8) and flow cytometry (FACS Calibur). All assays were conducted in triplicate.

### 2.10 SA‐β‐Gal Staining

The accumulation of SA‐β‐gal was assessed using a commercial kit (cat. no. C0602, Beyotime) following the manufacturer's instructions.

### 2.11 ABR Test

Mice were anesthetized with a mixture of 20 mg/kg tiletamine/zolazepam (Zoletil, Virbac, Carros, France) and 0.2 mg/kg dexmedetomidine (SML0956, Sigma‐Aldrich, USA). Body temperature was maintained stably during testing. ABRs were recorded using a TDT System III system (Tucker‐Davis Technologies, Alachua, FL, USA), with hearing thresholds tested at Click, 4, 8, 16, 24, and 32 kHz [16, 17].

### 2.12 Histological Immunofluorescence

Mouse temporal bones were collected, rinsed, and fixed in 4% paraformaldehyde for 2 h at room temperature. After decalcification in 10% EDTA (cat. no. G1105, Seville) for 24 h, cochleae were dissected into apical, middle, and basal turns. Tissues were permeabilized with 1% Triton X‐100 (cat. no. ST1722, Beyotime) for 2 h, blocked with 10% donkey serum (cat. no. D9663, Sigma) for another 2 h, and incubated overnight with primary antibodies: anti‐Myosin 7 (1:500, cat. no. 25–6790, Proteus BioSciences), anti‐NF (1:500, cat. no. AB5539, Sigma), Phalloidin (1:1000, cat. no. ab176753), and anti‐Ctbp2 (1:500, cat. no. 612044, BD BioSciences). Following washing, Alexa‐conjugated secondary antibodies were applied for 2 h at room temperature. Nuclei were counterstained with DAPI (1:10000, cat. no. 36308ES11, Yeasen). Images were captured using a Leica TCS SP8 confocal microscope and analyzed with ImageJ and Adobe Photoshop for hair cell quantification.

### 2.13 HE Staining

Cochlear tissue specimens were preserved in 4% paraformaldehyde at 4°C overnight, followed by decalcification in 10% EDTA for 48 h. The samples were then dehydrated sequentially in 15% and 30% sucrose solutions for 24 h each and subsequently embedded in optimal cutting temperature (OCT) compound. Frozen sections of approximately 10 μm in thickness were prepared from the cochlear samples and subjected to HE staining. Different regions (apical, middle, and basal turns) of the Stria vascularis (SV) were observed under a Nikon microscope (Tokyo, Japan). For SGN quantification, three sections were randomly selected from each cochlea/mouse, and all sections were examined at the same magnification.

### 2.14 Western Blotting

Samples were lysed in ice‐cold RIPA buffer supplemented with protease and phosphatase inhibitors for 30 min at 4°C and subsequently centrifuged at 12,000 × g for 20 min at 4°C. Protein concentrations in the supernatants were determined using a BCA protein assay kit. Equal amounts of protein were separated by SDS‐PAGE and transferred onto PVDF membranes (Merck Millipore, Darmstadt, Germany). After blocking with 5% non‐fat milk in TBST for 1 h at room temperature, the membranes were incubated overnight at 4°C with primary antibodies against Cleaved Caspase‐3 (1:1000, Cell Signaling Technology), phospho‐Beclin‐1 (1:1000, Cell Signaling Technology), LC3A/B (1:1000, Cell Signaling Technology), p62/SQSTM1 (1:1000, Cell Signaling Technology), PINK1 (1:1000, Cell Signaling Technology), eEF1A2 (1:1000, Abcam), GAPDH (1:5000, Proteintech), and β‐actin (1:5000, Proteintech). After washing with TBST, the membranes were incubated with the appropriate HRP‐conjugated secondary antibodies (1:5000) for 1 h at room temperature. Immunoreactive bands were visualized using a SuperSignal West Dura chemiluminescent substrate (Thermo Fisher Scientific, USA), and band intensities were quantified using ImageJ software (NIH, USA). All Western blot experiments were independently repeated at least three times.

### 2.15 RNA‐Seq Analysis

RNA was extracted using an RNeasy Mini Kit (Qiagen, Valencia, CA, USA). RNA purity, concentration, and integrity were evaluated with a NanoDrop 2000 (Thermo Fisher Scientific, USA) and Agilent 2100 Bioanalyzer (Agilent Technologies, Santa Clara, CA, USA), respectively. Libraries were constructed with the TruSeq Stranded mRNA LT Sample Prep Kit (Illumina, San Diego, CA, USA) and sequenced on an Illumina HiSeq × 10 platform (150 bp paired‐end reads). After trimming low‐quality reads with Trimmomatic, differential expression analysis was carried out using the DESeq R package. Genes with *p <* 0.01, fold change > 2, and FDR < 0.01 were considered differentially expressed. KEGG pathway enrichment was analyzed in *R* using the hypergeometric distribution.

### 2.16 Statistical Evaluations

All in vitro experiments were performed with at least three independent biological replicates. Sample sizes for animal experiments are indicated in the corresponding figure legends. Data normality was assessed using the Shapiro–Wilk test. Normally distributed data are presented as mean ± standard error of the mean (SEM), whereas non‐normally distributed data are presented as median with interquartile range (IQR). Comparisons among multiple groups were performed using one‐way ANOVA followed by Tukey's post hoc test for normally distributed data. For non‐normally distributed data, the Kruskal–Wallis test followed by Dunn's multiple‐comparisons test was used. Comparisons between two independent groups were performed using a two‐sided independent‐samples *t*‐test for normally distributed data or the Mann–Whitney *U* test for non‐normally distributed data. For repeated ABR measurements, two‐way repeated‐measures ANOVA followed by an appropriate multiple‐comparison correction was performed. Statistical analyses were conducted using GraphPad Prism 9, and *p* < 0.05 was considered statistically significant (*p* < 0.05, *p < 0.01, **p < 0.001; n.s., not significant).

## 3 Results

### 3.1 Long‐Term STB Administration Attenuates ARHL Progression in SAMP8 Mice

To evaluate the protective effects of STB on ARHL, we employed SAMP8 mice—a well‐established model of cochlear premature aging. Four‐week‐old male SAMP8 mice received 60 mg/kg STB via intragastric gavage every 2 days. An equivalent volume of vehicle was administered to the control group, with SAMR1 mice serving as the age‐matched control strain for SAMP8 senescent mice. ABR was measured at frequencies of Click, 4 kHz, 8 kHz, 16 kHz, 24 kHz, and 32 kHz at 2, 5, and 8 months of age (Figure 1A).

**FIGURE 1:**
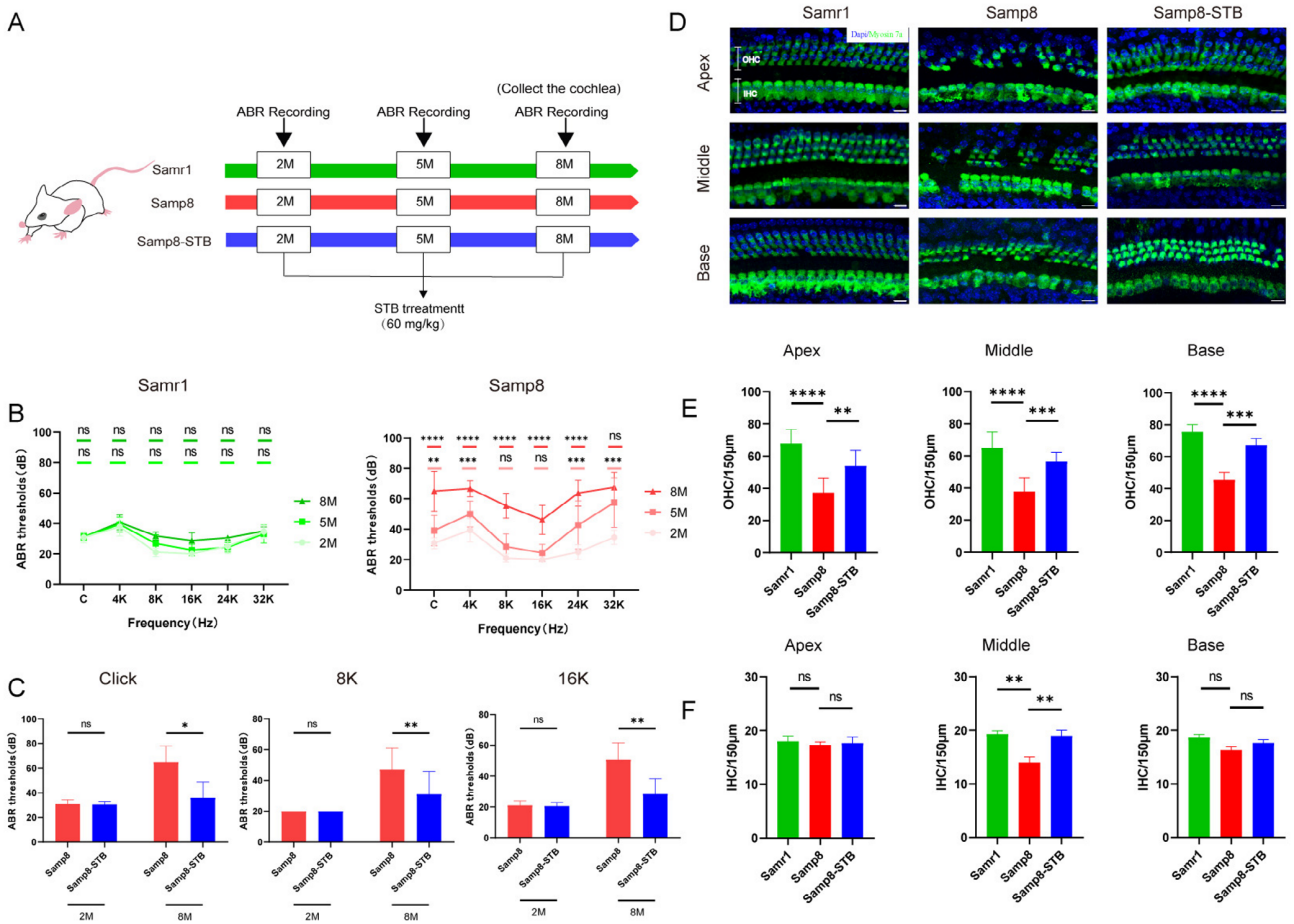
Effects of STB on ARHL in SAMP8 rapid aging model mice. (A) Experimental procedure. (B) ABR thresholds at click and 4, 8, 16, 24, 32 kHz were measured in SAMR1 mice(SAMR1, *n* = 8) and SAMP8 mice(SAMP8, *n* = 18) at 2, 5, and 8 months of age. (C) ABR thresholds at click,8 kHz and 16 kHz were measured in SAMP8 mice(SAMP8) and STB‐treated SAMP8 mice(SAMP8‐STB) at 2 and 8 months of age. (D) Immunofluorescence staining for Myosin 7a (green) and DAPI (blue) in the apical, middle, and basal turns of cochlear ducts from each group.(apical, *n* = 8, middle, *n* = 8, basal,*n* = 8). Scale bar = 20 μm. (E, F) Quantification of HC counts (labeled with Myosin 7a, green). Data are shown as mean ± SEM; For repeated ABR measurements, statistical analysis was performed using two‐way repeated‐measures ANOVA followed by an appropriate multiple‐comparison correction. Other comparisons between two independent groups were analyzed using a two‐sided independent‐samples *t*‐test.*p < 0.05, ***p < 0.001, ****p < 0.0001.

As shown in Figure 1B, ABR thresholds of the SAMP8 group at 5 months of age were significantly elevated with statistical difference at Click, 4 kHz, 24 kHz and 32 kHz compared with those at 2 months of age. At 8 months of age, the ABR thresholds of the SAMP8 group were all significantly increased with statistical difference at all tested frequencies including Click, 4 kHz, 8 kHz, 16 kHz, 24 kHz, and 32 kHz. In contrast, no significant elevation in ABR thresholds frequency was observed in the SAMR1 group across 2, 5, and 8 months of age, which confirms that aging induces hearing loss in SAMP8 mice. Subsequently, we compared the ABR thresholds between the SAMP8 control group and the STB‐treated group. As shown in Figure 1C, there were no significant differences in ABR thresholds at all tested frequencies between the two groups at 2 months of age, the STB‐treated group exhibited ABR thresholds significantly at 8 months of age, within click, 8 kHz and 16 kHz decreased 28.93 ± 5.08, 22.25 ± 2.78, and 22.25 ± 4.01 dB respectively. Nevertheless, there was no statistical difference between the STB‐treated group and the untreated group at 4 kHz, 24 kHz, and 32 kHz. These results indicate that long‐term STB administration can partially mitigate the progression of low‐frequency hearing loss in the SAMP8 accelerated aging model mice.

Following ABR testing, cochleae were collected, promptly fixed, and divided into apical, middle, and basal turns. HCs were labeled with Myosin7a antibody (Figure 1D). In the SAMP8 group, the number of Myosin7a‐positive outer hair cells (OHCs) was significantly reduced across all three turns compared to the SAMR1 group, with counts of 27.13 ± 4.56, 30.50 ± 4.40, and 30.00 ± 2.67 in the apical, middle, and basal turns, respectively; additionally, a statistically significant difference was also observed in the middle turn for inner hair cells (IHCs), with counts of 5.33 ± 0.66 respectively. However, long‐term STB administration starkly reduced OHC and middle turn IHC, compared to the untreated group which significantly reduced as 18.63 ± 3.56, 16.75 ± 4.58, 21.67 ± 2.58, and 5.00 ± 0.81 correspondingly. (Figure 1D,E).

Given that auditory signals are transmitted through synapses between IHCs and auditory neurons and aging is frequently associated with the loss of synapses and auditory neurons, the study further stained ribbon synapses of IHCs in the apical, middle and basal turns of the cochlea with anti‐Ctbp2 antibody (Figure 2A). The results showed that a significant reduction in the number of ribbon synapses in IHCs across all cochlear turns in the SAMP8 control group compared to the SAMR1 group which reduced as 12.58 ± 1.42, 11.58 ± 1.51, and 12.75 ± 1.31 significantly. However, long‐term administration of STB markedly mitigated synapse loss as 5.58 ± 1.43, 7.25 ± 1.40, and 6.75 ± 1.31 (Figure 2B). Subsequently, SGNs were stained with anti‐Tuj1 antibody and surviving SGNs were quantified (Figure 2C,D). Quantitative analysis of the apical, middle and basal turns in cochlears indicated a significant decrease in the mean density of SGNs in the SAMP8 control group relative to the SAMR1 group which significantly reduced as 26.40 ± 4.97,15.00 ± 0.81, and 22.67 ± 0.91 respectively, whereas the group receiving long‐term STB administration exhibited a significant increase in the number of surviving SGNs with 13.80 ± 5.48, 14.00 ± 4.61, and 10.00 ± 2.33 respectively compared with SAMP8 control group. These results indicate that long‐term STB administration may effectively reduce age‐related synapse loss.

**FIGURE 2:**
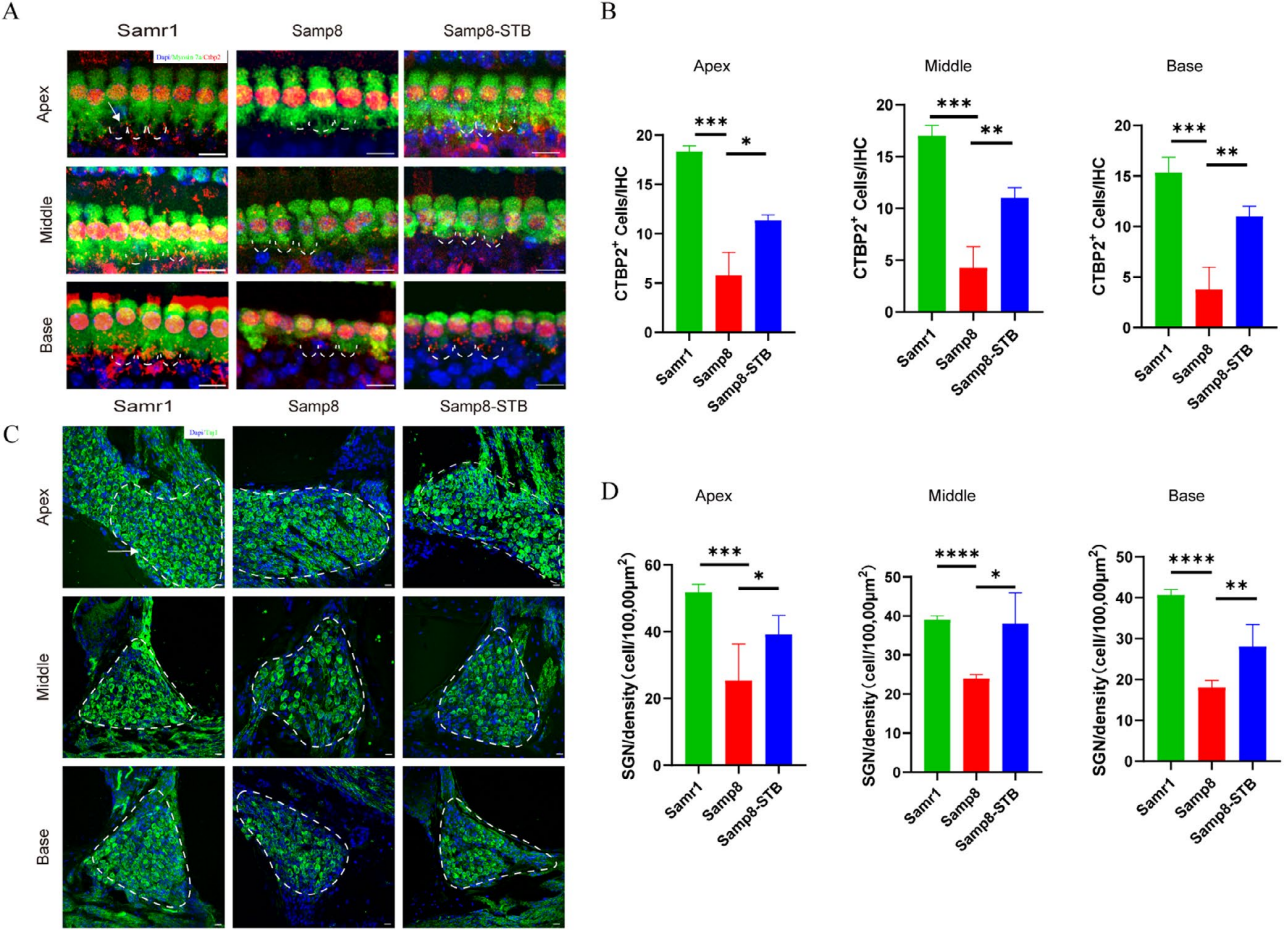
Effects of STB on ARHL in ribbon synapses and SGNs. (A) Immunofluorescence staining for Myosin 7a (green) and Ctbp2 (red) in the apical, middle and basal turns of cochlear ducts from each group. Scale bar = 20 μm. (B) Quantification of Ctbp2‐positive structures.(apical, *n* = 3, middle, *n* = 3, basal, *n* = 3) (C) Representative images of Tuj‐1 (green) staining for spiral ganglion neurons (SGNs) in the apical, middle, and basal turns of cochleae from each group. Scale bar = 20 μm. (D) Quantification of SGN counts (labeled with Tuj‐1, green). (apical, *n* = 5, middle, *n* = 5, basal, *n* = 5). Data are shown as mean ± SEM; statistical analysis was performed using two‐sided independent sample *t*‐test *p < 0.05, **p < 0.01, ***p < 0.001, ****p < 0.0001.

### 3.2 Long‐Term STB Administration Delays ARHL in C57BL/6J Mice

To further validate the protective effects of STB against ARHL observed in the SAMP8 model, we employed 12‐month‐old C57BL/6J mice as a naturally aging model, which is widely used to investigate age‐related auditory dysfunction. Compared with the accelerated‐aging SAMP8 model, C57BL/6J mice exhibit a more gradual decline in hearing function and provide a model that more closely reflects the progressive nature of ARHL during natural aging. Four‐month‐old C57BL/6J mice were used as young controls for comparison.

We then assessed STB's effect on auditory function in C57BL/6J mice using the same administration regimen as previous in vivo studies (60 mg/kg by alternate‐day intragastric gavage) (Figure 3A). Our findings revealed that continuous administration of STB over a six‐month period decelerated the progression of ARHL in C57BL/6J mice at click and 8 kHz frequencies, although no significant improvement was observed at higher frequencies. A comparison of hearing threshold shifts indicated that the STB‐treated group exhibited smaller increases in ABR thresholds than the untreated control group at click and 8 kHz (Figure 3B,C). These findings are consistent with results obtained from the SAMP8 mouse model, suggesting that protective effects of STB against ARHL are predominantly confined to mid and low frequencies and are not specific to a particular strain.

**FIGURE 3:**
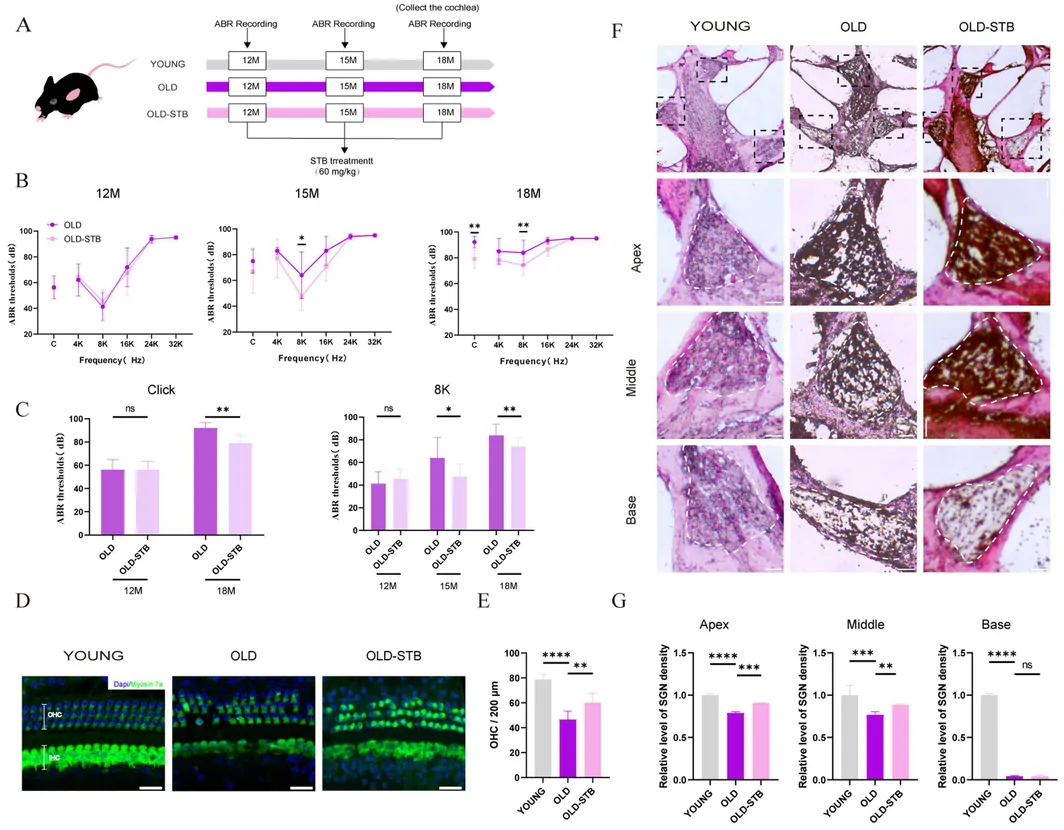
Effects of STB on age‐related hearing loss in C57 mice. (A) Experimental procedure. (B, C) ABR thresholds at click and 4, 8, 16, 24, 32 kHz were measured in 12‐month‐old C57 mice (OLD, *n* = 12) and STB‐treated 12‐month‐old C57 mice (OLD‐STB, *n* = 12) at baseline, 3 months and 6 months of observation. (D) Immunostaining for Myosin7a (green) and DAPI (blue) in the middle turns of cochleae from different groups. Scale bar = 20 μm (E) Quantification of HC counts (labeled with Myosin7a, green) (*n* = 8). (F) Representative images of cochlear HE staining. Scale bar = 20 μm (G) Relative number of SGNs in the OLD and OLD‐STB groups compared to the YOUNG group. (each group, *n* = 6). Data are shown as mean ± SEM; For repeated ABR measurements, statistical analysis was performed using two‐way repeated‐measures ANOVA followed by an appropriate multiple‐comparison correction. Other comparisons between two independent groups were analyzed using a two‐sided independent‐samples *t*‐test. ***p < 0.001, ****p < 0.0001.

After ABR testing, cochlear tissues were fixed and dissected, and hair cell survival was assessed by Myosin7a immunostaining. (Figure 3D). Analysis of hair cell counts indicated that prolonged administration of STB significantly mitigated the loss of OHCs in the middle turn of the cochlea which reduced as 13.60 ± 3.17 respectively (Figure 3E), suggesting that the effect of STB in delaying cochlear hair cell loss is not specific to any particular strain. Subsequently, we investigated the protective effects of STB on SGNs in aged C57BL/6J mice using frozen sectioning and hematoxylin and eosin (HE) staining (Figure 3F,G). Our findings revealed that long‐term STB administration significantly attenuated the reduction in relative SGN density in the apical and middle turns of the cochlea, with decreases limited to 0.09 ± 0.00‐fold and 0.23 ± 0.07‐fold, respectively. Collectively, our results demonstrate that STB delays the progression of ARHL in the C57BL/6J mouse model, corroborating similar findings in the SAMP8 mouse model. These data further substantiate that the protective effect of STB against ARHL is not strain‐specific.

### 3.3 STB Delays D‐Gal‐Induced Age‐Related Damage in HCs and SGNs

To further evaluate the protective effects of STB against inner ear aging, we initially established an in vitro aging model. Cochlear explants isolated from neonatal ICR mice were incubated with 60 mg/mL D‐gal [18, 19]. In comparison with the vehicle control, D‐gal‐induced damage resulted in a 1.73 ± 0.12‐fold increase in senescence‐associated SA‐β‐gal activity after 72 h (Figure 4A,B), with SA‐β‐gal being widely recognized as a marker of aging. Moreover, concurrent treatment with 60 μM STB significantly reversed the D‐gal‐induced increase in SA‐β‐gal activity compared with the D‐gal‐treated group. (0.833 ± 0.133‐fold, *p* = 0.003). These findings suggest that STB effectively delays the aging process of the cochlea in vitro.

**FIGURE 4:**
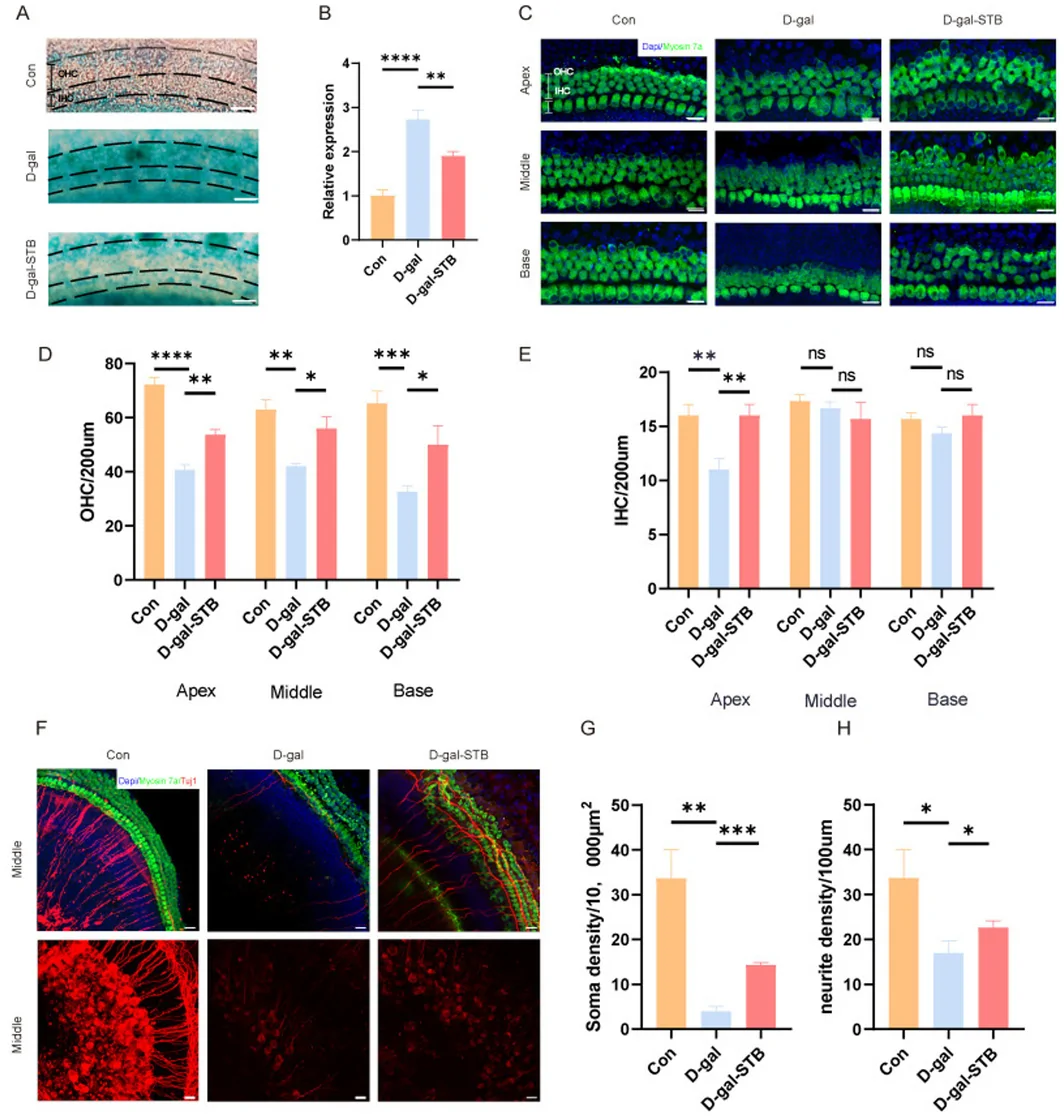
Effects of STB on D‐gal‐induced damage in cochlear explants. (A) Senescence‐associated β‐galactosidase (SA‐β‐gal) staining (blue). Scale bar = 40 μm. (B) Relative expression levels of SA‐β‐gal in each group (each group, *n* = 6). (C) Confocal images of cochlear explants (apical, middle, and basal turns) from each treatment group [control (Con), D‐gal, D‐gal + STB (STB‐D‐gal)] after 24 h treatment, immunostained for Myosin 7a (green) and DAPI (blue). Scale bar = 20 μm. (D, E) Quantification of hair cell counts (based on Myosin 7a green fluorescence; each group, *n* = 6). (F) Effects of STB on D‐gal‐induced cochlear SGNs injury in vitro. Immunofluorescence staining for Tuj‐1 (red) and Myosin 7a (green) in the apical turns of cochleae from each group. Scale bar = 20 μm (G, H) Quantification analysis of SGN soma density and neurite density in the apical turns of cochlear explants for all treatment conditions (each group, *n* = 12). Data are shown as mean ± SEM; statistical analysis was performed using two‐sided independent sample *t*‐test; *p < 0.05, ***p < 0.001.

To further investigate the protective effects of STB against D‐gal‐induced cochlear injury, immunostaining for Myosin 7a and Tuj1 was performed to assess hair cell and SGN loss. As shown in (Figure 4C–E)‐gal treatment significantly reduced OHC numbers in the apical (31.67 ± 1.88, *p* < 0.001), middle (1.52, *p* = 0.005), and basal turns (32.67 ± 2.86, *p* < 0.001), as well as IHC numbers in the apical turn (5.00 ± 0.82, *p* = 0.003), compared with the vehicle group. Co‐treatment with STB significantly increased OHC numbers in the apical (13.00 ± 1.70, *p* = 0.001), middle (2.52, *p* = 0.03), and basal turns (17.33 ± 4.22, *p* = 0.014), compared with the D‐gal group. Similarly, STB treatment significantly increased IHC numbers in the apical turn (5.00 ± 0.81, *p* = 0.003) compared with the D‐gal group. As shown in (Figure 4D,F,G)‐gal treatment significantly reduced neurite and SGN soma densities by 29.67 ± 3.71 (*p* = 0.001) and 16.67 ± 3.97 (*p* = 0.013), respectively, compared with the vehicle group, whereas STB co‐treatment significantly restored these reductions, with increases of 10.33 ± 0.67 and 5.67 ± 1.76, respectively, relative to the D‐gal group. These findings indicate that STB treatment can significantly alleviate the senescence of HCs and SGNs in the D‐gal‐induced in vitro cochlear aging model.

### 3.4 RNA‐Seq Identifies STB‐Mediated Reversal of the eEF1A2‐Beclin 1 Pathway in D‐Gal‐Induced Cellular Model

To investigate the biological mechanisms underlying the protective effects of STB against ARHL, we developed an aging model using HEI‐OC1 cells. The HEI‐OC1 cells were exposed to D‐gal at concentrations of 10, 20, 25, 30, and 40 mg/mL for 72 h, resulting in a dose‐dependent decrease in cell viability (Figure 5A). A concentration of 20 mg/mL D‐gal, which led to a 26.6% ± 4.35% reduction in cell viability, was subsequently selected for further experiments. Following this, HEI‐OC1 cells were co‐treated with STB at concentrations of 20, 25, 30, and 50 μM (Figure 5B). A concentration of 25 μM STB, which resulted in a 27.82% ± 2.88% increase in cell viability compared to D‐gal‐induced damage alone, was chosen for continued study. Furthermore, β‐galactosidase staining indicated that STB co‐treatment significantly reduced senescence‐associated β‐galactosidase activity compared to the D‐gal group (Figure 5C,D). Collectively, these findings suggest that STB can mitigate D‐gal‐induced senescence in HEI‐OC1 cells.

**FIGURE 5:**
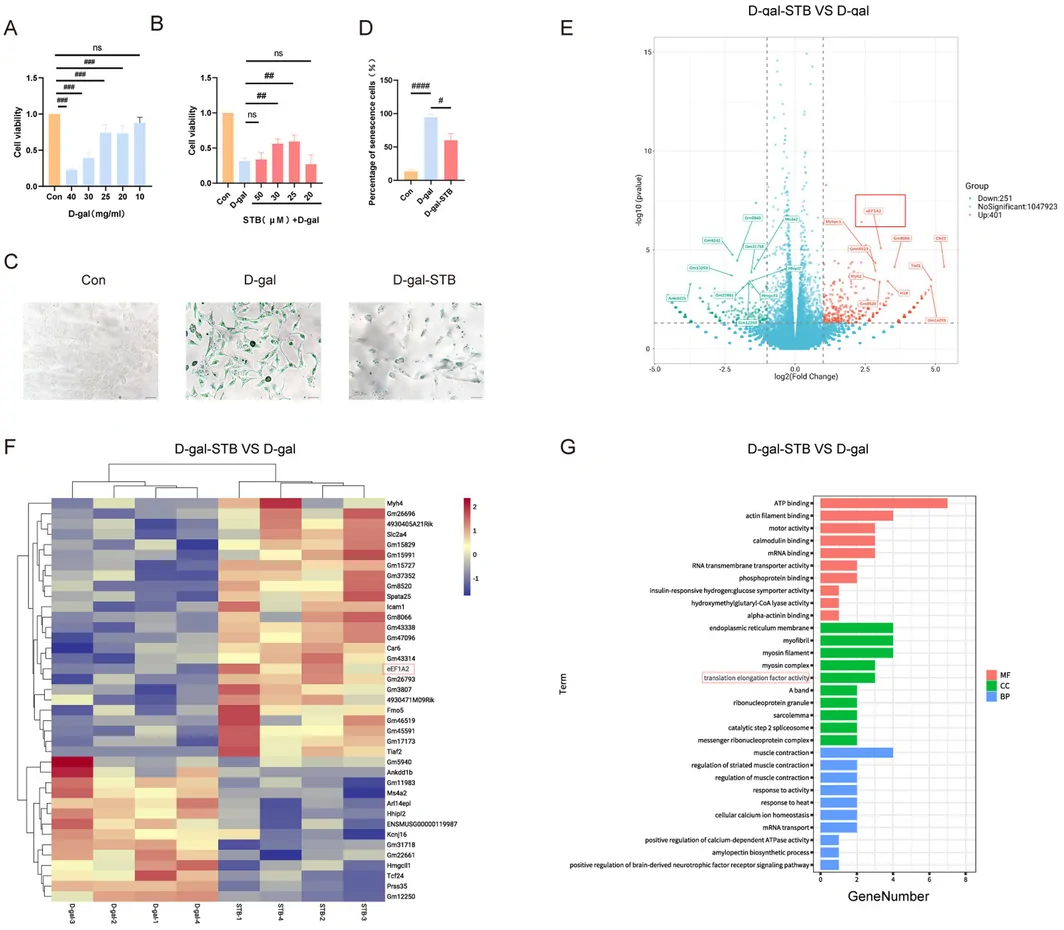
Effects of STB on transcriptomic profiles of D‐gal‐induced senescent HEI‐OC1 cells. (A) Effect of D‐gal on HEI‐OC1 cell viability detected by CCK‐8 assay (each group, *n* = 4). (B) Effect of different concentrations of STB on HEI‐OC1 cell viability after STB + D‐gal‐induced damage evaluated by CCK‐8 assay (each group, *n* = 4). (C, D) SA‐β‐gal staining results. Scale bar = 20 μm. (E) Volcano plot showing differentially expressed genes (DEGs) between D‐gal group and STB—D‐gal group. (F) Heatmap of DEGs in HEI‐OC1 cells between D‐gal group and STB—D‐gal group. (G) Gene Ontology (GO)‐based gene function analysis showing the affected pathways after STB treatment in HEI‐OC1 cells. Data are shown as mean ± SEM; statistical analysis was performed using two‐sided independent sample *t*‐test; ***p < 0.001, ****p < 0.0001.

Subsequently, we performed RNA sequencing to compare the transcriptomic profiles of the D‐gal group and the STB–D‐gal co‐treatment group in this cellular senescence model. Using the differential expression criteria of adjusted *p* < 0.01, FDR < 0.01, and |fold change| > 2, we identified 401 upregulated and 251 downregulated genes in the STB–D‐gal co‐treatment group relative to the D‐gal group. The distribution and expression patterns of these differentially expressed genes are illustrated by the volcano plot (Figure 5E) and heatmap (Figure 5F). GO enrichment analysis (Figure 5G) revealed 30 significantly enriched biological processes, including cellular metabolism, signal transduction, and stress responses. Among these differentially expressed genes, eEF1A2 exhibited a significant increase in expression following STB treatment. This differential expression pattern suggests that eEF1A2 may contribute to the protective effects of STB against D‐gal‐induced cellular senescence.

### 3.5 STB Upregulates eEF1A2 Expression and Promotes Autophagy in HCs

Autophagy dysfunction has been identified as a significant contributor to hearing loss. To investigate whether eEF1A2 modulates the expression of genes in the autophagy pathway, we used Western blotting to assess the protein levels of eEF1A2 and its downstream autophagy‐related protein Beclin‐1, a key regulator of autophagy initiation. As shown in (Figure 6A,C,D)‐gal treatment significantly decreased eEF1A2 and Beclin‐1 protein levels by 0.40 ± 0.12‐ and 0.66 ± 0.06‐fold, respectively, compared with the control group. Whereas STB treatment reversed these reductions by 0.33 ± 0.04‐ and 0.32 ± 0.03‐fold, respectively, relative to the D‐gal group. To further evaluate the effect of STB on autophagy, we examined the protein levels of LC3‐II and p62. As shown in (Figure 6D,E)‐gal treatment reduced LC3‐II levels to 0.63 ± 0.04‐fold of control levels and increased p62 levels by 0.22 ± 0.06‐fold compared with the control group, whereas STB treatment reversed these changes, increasing LC3‐II by 0.45 ± 0.03‐fold and decreasing p62 by 0.32 ± 0.07‐fold compared with the D‐gal group.

**FIGURE 6:**
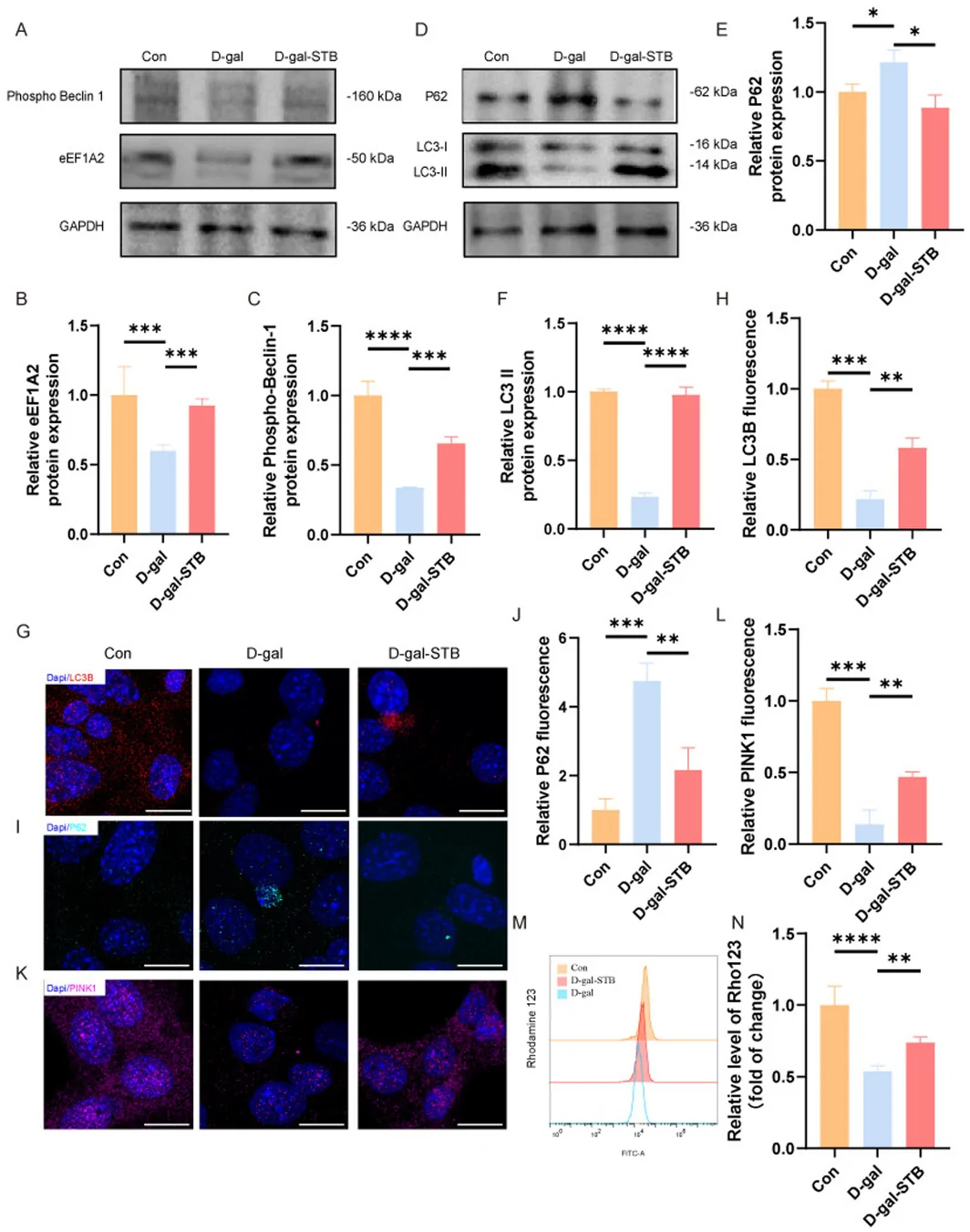
Effects of STB on eEF1A2 expression and autophagy‐related pathways in D‐gal‐induced senescent HEI‐OC1 cells. (A–F) Relative expression levels and protein quantitative analysis of eEF1A2, Phospho‐Beclin‐1, LC3II and P62 detected by Western blot. (each group, *n* = 3). (G, H) Representative images and quantitative analysis of LC3B protein in HEI‐OC1 cells of each group. (each group, *n* = 3). Scale bar = 20 μm. (I, J) Representative images and quantitative analysis of P62 in HEI‐OC1 cells of each group. (each group, *n* = 3). Scale bar = 20 μm (K, L) Representative images and quantitative analysis of PINK1 in HEI‐OC1 cells of each group. (each group, *n* = 3). Scale bar = 20 μm (M, N) Representative images and quantitative analysis of Rhodamine 123 (for mitochondrial membrane potential detection) in HEI‐OC1 cells of each group. Data are presented as mean ± SEM; statistical analysis was performed using two‐sided independent sample *t*‐test; **p < 0.01, ***p < 0.001, ****p < 0.0001.

To further examine the effects of STB on autophagy‐related changes, immunofluorescence staining was performed to evaluate the fluorescence intensities of LC3B, p62, and PINK1 that have recently been widely used to analyze autophagic flux(Figure 6G–L). The results showed that D‐gal treatment decreased LC3B and PINK1 fluorescence intensities by 0.78 ± 0.04‐ and 0.86 ± 0.07‐fold, respectively, and increased p62 fluorescence intensity by 3.74 ± 0.35‐fold compared with the control group. STB treatment reversed these D‐gal‐induced changes, increasing LC3B and PINK1 fluorescence intensities by 0.36 ± 0.05‐ and 0.33 ± 0.06‐fold, respectively, and decreasing p62 fluorescence intensity by 2.58 ± 0.48‐fold relative to the D‐gal group. As a reduction in mitochondrial membrane potential (ΔΨm) is associated with mitochondrial dysfunction and mitophagy, ΔΨm was further assessed using Rhodamine 123. As shown in (Figure 6D,M,N)‐gal exposure for 72 h reduced Rhodamine 123 fluorescence to 0.46 ± 0.08‐fold of the control level, whereas STB treatment partially reversed this reduction, increasing the normalized fluorescence by 0.20 ± 0.03‐fold relative to the D‐gal group.

### 3.6 STB Protects HCs Against Age‐Related Apoptosis

Given that impaired autophagy is associated with increased apoptosis, we examined the protein level of Cleaved Caspase‐3 using Western blotting(Figure 7A,B). The results showed that D‐gal treatment increased Cleaved Caspase‐3 protein levels by 2.14 ± 0.13‐fold compared with the control group, whereas STB treatment reversed this increase, reducing Cleaved Caspase‐3 levels by 0.44 ± 0.11‐fold relative to the D‐gal group. Consistent with the western blotting results, immunofluorescence analysis showed that STB treatment significantly reduced Cleaved Caspase‐3 expression by 14.55 ± 0.82‐fold compared with the D‐gal group (Figure 7C,D).

**FIGURE 7:**
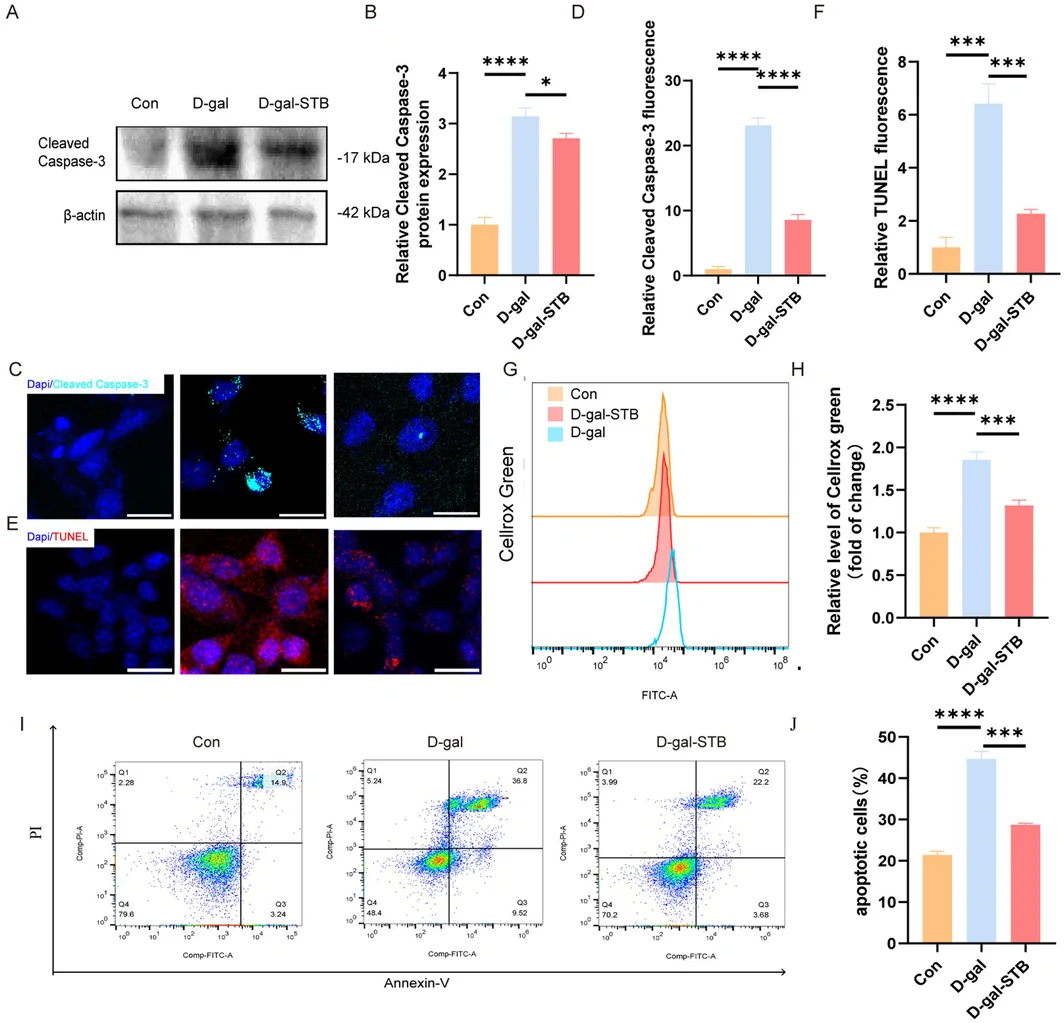
Effects of STB on Apoptosis in D‐gal‐induced Senescent HEI‐OC1 Cells (A‐B) Relative expression levels and protein quantitative analysis of Cleaved Caspase‐3 detected by Western blot. (each group, *n* = 3).(C, D) Representative images and quantitative analysis of Cleaved Caspase‐3 in HEI‐OC1 cells of each group. (each group, *n* = 3). Scale bar = 20 μm (E, F) Quantification of TUNEL‐positive cells normalized to the total number of cells. Scale bar = 20 μm. (each group, *n* = 3). (G, H) Representative flow cytometry plots and quantification of apoptotic cells detected using an Annexin V‐FITC/PI apoptosis detection kit. (I, J) Representative images and quantitative analysis of CellRox Green staining (for total ROS detection) in HEI‐OC1 cells of each group. Data are presented as mean ± SEM; statistical analysis was performed using two‐sided independent sample *t*‐test; **p < 0.01, ***p < 0.001, ****p < 0.0001.

Furthermore, TUNEL staining was performed to evaluate apoptosis in HEI‐OC1 cells (Figure 7E,F). D‐gal treatment increased TUNEL positivity by 5.424 ± 0.482‐fold compared with the control group, whereas STB treatment reversed this increase, reducing TUNEL positivity by 4.16 ± 0.44‐fold relative to the D‐gal group.

Given the involvement of mitochondrial ROS in STB‐mediated protection of hair cells, we conducted further investigations to determine whether STB could enhance mitochondrial function in senescent hair cells. Flow cytometric analysis using CellROX Green showed that D‐gal treatment increased ROS accumulation by 0.86 ± 0.06‐fold compared with the control group (Figure 7G,H), whereas STB treatment reversed this increase, reducing ROS levels by 0.54 ± 0.06‐fold relative to the D‐gal group. Apoptosis was further assessed by Annexin V‐FITC/PI flow cytometry. As shown in (Figure 7D,I,J)‐gal treatment increased the apoptotic cell percentage to 23.21% ± 1.16% compared with the control group, whereas STB treatment reduced it to 15.95% ± 1.08% compared with the D‐gal group.

### 3.7 Inhibition of Autophagy or eEF1A2 Attenuates the Protective Effects of STB in a Cellular Senescence Model

Although our study showed that STB promotes eEF1A2 expression and induces autophagy, the relationship between these two effects remains unclear. To further investigate the role of autophagy in the protective effects of STB, D‐galactose‐induced senescent HEI‐OC1 cells were treated with STB in the presence or absence of the autophagy inhibitor 3‐methyladenine (3‐MA). The protein level of LC3‐II was then examined by Western blotting. As shown in (Figure 8B), STB treatment significantly increased LC3‐II protein expression in D‐galactose‐induced senescent HEI‐OC1 cells, resulting in a 0.60 ± 0.04‐fold elevation. However, 3‐MA treatment markedly attenuated this increase, reducing LC3‐II levels to 0.25 ± 0.03‐fold relative to the STB‐treated group (Figure 8C).

**FIGURE 8:**
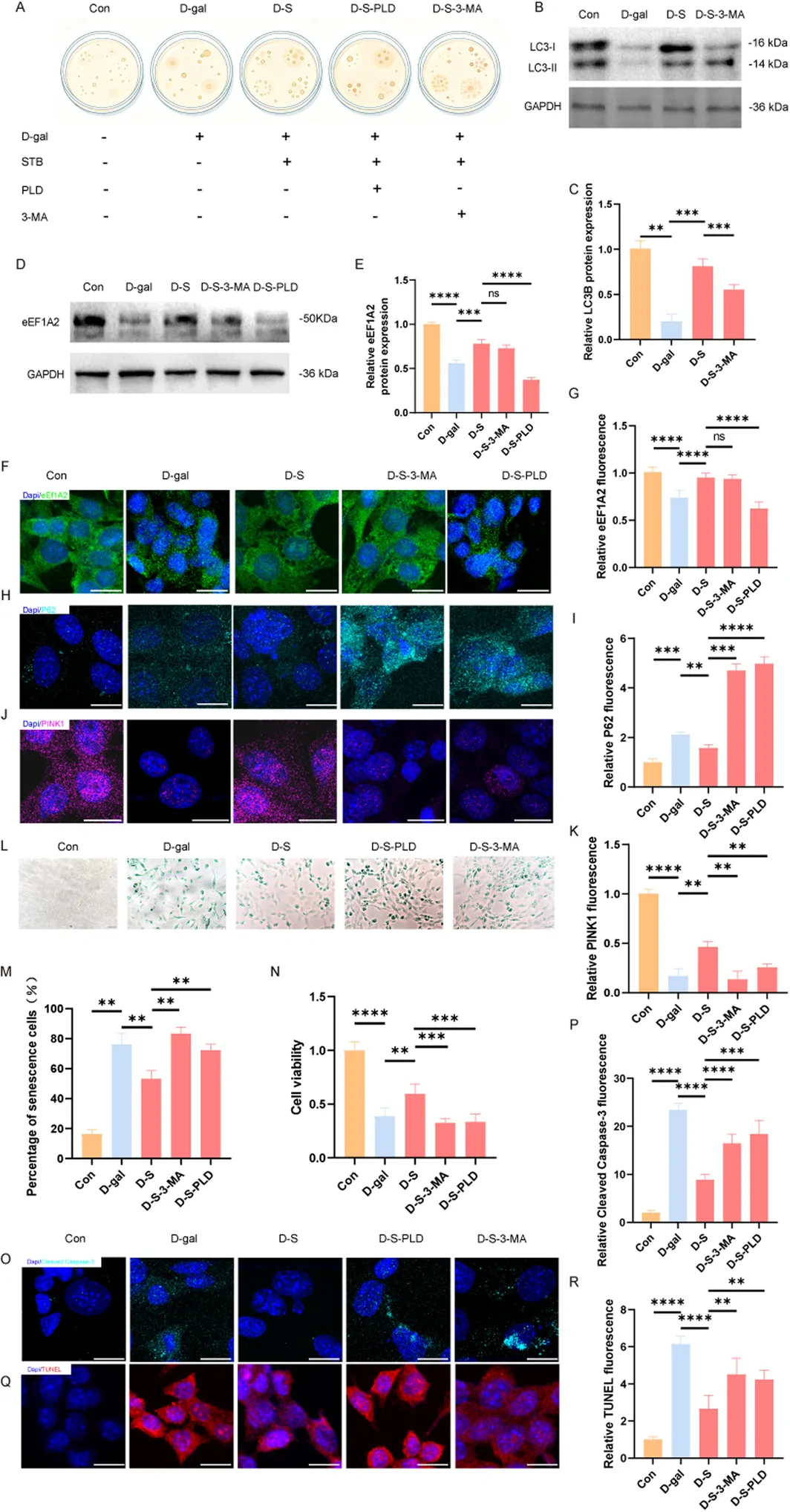
Inhibiting Autophagy or eEF1A2 Reverses the Protective Effect of STB on HCs in the Aging Model (A) Experimental procedure. (B, C) Relative expression levels and protein quantitative analysis of LC3‐II detected by Western blot. (D, E) Relative expression levels and protein quantitative analysis of eEF1A2 detected by Western blot. (F–K) Representative immunofluorescence images and quantification of eEF1A2 (F, G), P62 (H, I), and PINK1 (J, K) expression in HEI‐OC1 cells from each group. (*n* = 3 independent biological replicates). Scale bar = 20 μm. (L, M) SA‐β‐gal staining results and relative expression levels of SA‐β‐gal in each group. (each group, *n* = 3). Scale bar = 20 μm. (N) CCK‐8 assay showing the viability of HEI‐OC1 cells treated with D‐gal and STB in the presence or absence of 3‐MA or PLD. (each group, *n* = 3). Scale bar = 20 μm. (O, P) Representative images and quantitative analysis of Cleaved Caspase‐3 in HEI‐OC1 cells of each group. (each group, *n* = 3). Scale bar = 20 μm. (Q, R) Quantification of TUNEL‐positive cells normalized to the total number of cells. Scale bar = 20 μm. (each group, *n* = 3). Data are presented as mean ± SEM; statistical analysis was performed using two‐sided independent sample *t*‐test; **p < 0.01, ***p < 0.001, ****p < 0.0001.

To further investigate whether eEF1A2 is involved in STB‐associated autophagy regulation, senescent HEI‐OC1 cells were treated with STB, 3‐MA, or Plitidepsin (PLD), an eEF1A2‐targeting inhibitor. Western blotting showed that 3‐MA treatment did not significantly alter eEF1A2 expression compared with the STB‐treated group (Figure 8D), whereas PLD treatment significantly reduced eEF1A2 protein levels to 0.40 ± 0.02‐fold relative to the STB‐treated group (Figure 8E). Consistent with the Western blotting results, immunofluorescence analysis further confirmed that PLD treatment decreased eEF1A2 expression to 0.32 ± 0.04‐fold compared with the STB‐treated group(Figure 8F,G). We further examined the autophagy‐related proteins p62 and PINK1 by immunofluorescence staining (Figure 8H,J). Compared with the STB‐treated group, both 3‐MA and PLD treatments significantly increased p62 expression by 3.12 ± 0.17‐fold and 3.41 ± 0.18‐fold, respectively, while decreasing PINK1 expression to 0.32 ± 0.05‐fold and 0.20 ± 0.03‐fold, respectively (Figure 8I,K). Taken together, these results demonstrate that inhibition of autophagy or eEF1A2 suppresses STB‐associated autophagic responses, suggesting that eEF1A2 is involved in the regulation of autophagy during STB‐mediated protection in senescent HEI‐OC1 cells.

We further assessed cellular senescence and viability. β‐galactosidase staining showed that, compared with the STB‐treated group, 3‐MA treatment partially abolished the inhibitory effect of STB on β‐galactosidase activity, resulting in a 29.99% ± 2.89% increase. Similarly, PLD treatment produced a comparable effect, increasing β‐galactosidase activity by 19.08% ± 2.81% relative to the STB‐treated group(Figure 8L,M). CCK‐8 assays further showed that both 3‐MA and PLD treatments significantly reduced the STB‐mediated improvement in cell viability, decreasing cell viability to 0.27 ± 0.02‐fold and 0.26 ± 0.03‐fold, respectively, compared with the STB‐treated group(Figure 8N).

We next assessed apoptosis by immunofluorescence staining for Cleaved Caspase‐3. Compared with the STB‐treated group, treatment with either 3‐MA or PLD significantly increased Cleaved Caspase‐3 expression by 3.41 ± 0.18‐fold and 9.48 ± 1.35‐fold, respectively, indicating a reversal of the anti‐apoptotic effect of STB(Figure 8O,P). Consistently, TUNEL staining showed that 3‐MA and PLD treatments increased TUNEL‐positive cells by 1.85 ± 0.45‐fold and 1.57 ± 0.35‐fold, respectively, relative to the STB‐treated group(Figure 8Q,R).

Taken together, these findings indicate that inhibition of autophagy or eEF1A2 attenuates the protective effects of STB against cellular senescence and apoptosis in HEI‐OC1 cells, suggesting that eEF1A2 and autophagy contribute to STB‐mediated cytoprotection.

## 4 Discussion

ARHL ranks among the most common neurodegenerative conditions globally, predominantly affecting older adults [20]. In this study, we systematically examined the protective effects and mechanisms of STB against ARHL using complementary in vitro and in vivo models. STB attenuated D‐gal‐induced senescence in cochlear explants and HEI‐OC1 cells, reduced hearing threshold shifts, and alleviated cochlear degeneration in both SAMP8 and naturally aged C57BL/6J mice. Mechanistically, STB targeted the eEF1A2‐Beclin 1 pathway to enhance mitophagy, reduce oxidative stress, and inhibit caspase‐3‐dependent apoptosis. These findings highlight STB as a promising natural candidate for ARHL intervention.

Previous studies have reported that several natural compounds, including NMN, resveratrol, and baicalin [21] exert protective effects against ARHL mainly via improving mitochondrial function, activating SIRT1‐mediated autophagy [22], or inhibiting inflammatory and oxidative stress pathways [23]. However, few studies have explored the therapeutic potential of lignans derived from *Schisandra chinensis* in age‐related cochlear degeneration. Our study is the first to demonstrate that STB significantly alleviates cellular senescence and degeneration in auditory cells under aging‐related stress. In D‐galactose‐induced in vitro aging models, STB pretreatment reduced the number of SA‐β‐gal‐positive cells, improved cell viability, and maintained the structural integrity of cochlear hair cells and neurons. These results suggest that STB possesses strong anti‐senescent and cytoprotective properties in the inner ear, which are consistent with its well‐documented antioxidant, anti‐inflammatory, and mitochondrial‐protective activities in other tissues [24, 25]. STB modulated multiple pathological processes involved in ARHL, including oxidative stress, mitophagy, and apoptosis, producing more comprehensive and sustained protective effects [8].

In this study, STB demonstrated consistent protective efficacy in two well‐established ARHL models: accelerated‐senescence SAMP8 mice and naturally aged C57BL/6J mice [26, 27, 28]. Long‐term oral STB significantly alleviated low‐to‐mid frequency hearing loss and attenuated cochlear hair cell, synaptic, and spiral ganglion neuron degeneration in both SAMP8 and aged C57BL/6J mice. The consistent efficacy across accelerated and natural aging models supports the robustness and translational potential of STB's otoprotective effects. Mechanistically, RNA‐seq revealed 251 downregulated and 401 upregulated genes (D‐gal+STB vs. D‐gal), with heatmap clustering highlighting differential expression of autophagy and mitochondrial‐related genes, including Myh4, Slc2a, and Gm26696. GO enrichment analysis revealed that STB treatment significantly modulated pathways related to ATP binding, actin filament binding, and translation elongation factor activity, with eEF1A2 (a translation elongation factor) emerging as a key candidate.

STB protects against cochlear aging by regulating the eEF1A2/Beclin‐1 pathway to restore mitochondrial function, reduce oxidative stress, and inhibit senescence. Mechanistically, STB upregulates eEF1A2, which enhances Beclin‐1‐dependent mitophagy, limits ROS accumulation, and suppresses caspase‐3‐mediated apoptosis. These findings reveal a novel molecular cascade underlying the protective effects of STB against cochlear aging.

Accumulating evidence confirms that impaired mitophagy and mitochondrial dysfunction are core pathogenic events in ARHL development and progression [29]. Under aging conditions, failure to clear dysfunctional mitochondria leads to sustained ROS overproduction, oxidative stress, chronic inflammation, and eventual apoptosis of cochlear hair cells and spiral ganglion neurons [30]. By restoring mitophagy, STB preserves mitochondrial homeostasis, reduces oxidative stress and apoptosis in the aging inner ear, and protects cochlear structure and auditory function. This eEF1A2‐Beclin 1‐mediated mechanism expands current understanding of natural product protection against ARHL, although eEF1A2 is known to function in protein synthesis, mitochondrial regulation, and neuronal protection in other systems [31, 32]., its specific role in ARHL and its regulation by natural compounds have rarely been reported. Our findings therefore provide novel mechanistic insights into the protective effects of traditional Chinese medicine‐derived components against ARHL and highlight the eEF1A2‐Beclin 1 signaling pathway as a promising and innovative therapeutic target for future drug development. The consistent protective efficacy across multiple ARHL models and the newly identified eEF1A2‐Beclin‐1‐mediated mitophagy pathway make STB a highly promising natural candidate for the prevention and intervention of ARHL. These results enrich the pharmacological profile of *Schisandra chinensis*‐derived components, providing a solid scientific foundation for the translational development of STB as a potential therapeutic agent for clinical use in elderly individuals with ARHL.

Nevertheless, this study has several limitations. First, the long‐term efficacy and safety of STB remain to be established, as ARHL progresses throughout the lifespan. Whether prolonged STB administration can provide sustained auditory protection at more advanced ages therefore warrants further investigation. Second, although pharmacological inhibition of autophagy with 3‐MA and inhibition of eEF1A2 with PLD attenuated the protective effects of STB, supporting the involvement of eEF1A2 and autophagy in STB‐mediated protection, these findings do not establish a direct causal relationship between eEF1A2 and autophagy/mitophagy. In particular, whether eEF1A2 directly regulates Beclin‐1‐associated autophagic signaling remains to be determined. Further studies employing genetic loss‐ and gain‐of‐function approaches, together with rescue experiments, are therefore required to clarify the mechanistic relationship among eEF1A2, Beclin‐1, and mitophagy and to validate this regulatory mechanism in vivo. Finally, only male mice were used to minimize sex‐related variability. Given the reported sex differences in ARHL progression, future studies including both sexes are needed to determine whether the protective effects of STB are sex‐dependent.

In conclusion, STB attenuates age‐related cochlear degeneration and hearing loss by enhancing eEF1A2/Beclin‐1‐mediated mitophagy. Its consistent protective effects across in vitro and in vivo aging models support STB as a promising natural candidate for ARHL prevention and treatment.

## 5 Conclusions

This study demonstrates that STB delays ARHL progression and protects against age‐related auditory damage, with eEF1A2 and autophagy contributing to its protective effects. These findings support STB as a potential therapeutic candidate for ARHL and provide experimental evidence for the further investigation and application of traditional Chinese medicine in age‐related hearing disorders.

## Author Contributions

Gongrui Tang, Tianhong Zhang, Yilai Shu and Ziwen Gao conducted the study design. Gongrui Tang, Ziwen Gao., Tianhong Zhang, Yilai Shu, Tianyu Ma., Mengting Liu, Jiangping Xie, Ping yang, and Tianhong Zhang performed data analysis, interpretation, and manuscript preparation. Gongrui Tang, Ziwen Gao, Jiangping Xie performed biochemical experiments and data collection. Gongrui Tang, Ziwen Gao., Tianyu Ma., Mengting Liu, Jiangping Xie conducted animal behavioral tests. AGongrui Tang, Ziwen Gao sur‐geries and performed immunohistochemical quantification. Gongrui Tang and Ziwen Gao, Ping yang, provided guidance and resources for analysis. All authorsreviewed and approved the final version of the manuscript.

## Funding

The authors thank the Core Facility of Shanghai Medical College, Fudan University and Harbin Medical University, China. This work was funded by National Key Research and Development Program of China (2023YFC2412105 to Tianhong Zhang); Heilongjiang Provincial Key Research and Development Program (2023ZX06C07 to Tianhong Zhang); The authors thank the Core Facility of Shanghai Medical College, Fudan University, China. This work was funded by the National Natural Science Foundation of China (grants 82225014, 82521001, 82225014, 82192864 to YLS); Shanghai Municipal Health Commission (grant 2024CXJQ02 to YLS).

## Ethics Statement

All animal procedures were approved by the IACUC of Fudan University and the First Affiliated Hospital of Harbin Medical University (No: 202410065S; IACUC‐2023092).

## Conflicts of Interest

The authors declare no conflicts of interest.

## Supporting information


**Figure S1:** Dose–response evaluation of STB treatment in SAMP8 mice. (A) Experimental design for evaluating the protective effects of different doses of STB in 2‐month‐old SAMP8 mice. Mice were randomly divided into four groups: control group, STB 10 mg/kg group, STB 60 mg/kg group, and STB 100 mg/kg group (*n* = 6 per group). STB was administered every other day for 3 months. (B) ABR thresholds at Click and 4, 8, 16, 24, and 32 kHz were measured before treatment and after 3 months of STB administration. Data are presented as mean ± SEM. Statistical analysis was performed using two‐way repeated‐measures ANOVA followed by appropriate multiple‐comparison correction. *p* < 0.05.

## Data Availability

The data that support the findings of this study are available on request from the corresponding author. The data are not publicly available due to privacy or ethical restrictions.
