# Supplementary material for: Schisantherin B Preserves Cochlear Function and Hair Cell Integrity in Aging by Restoring Mitochondrial Homeostasis via eEF1A2–Beclin‐1–Mediated Mitophagy
Source: CNS Neurosci Ther. 2026 Nov 13;32(11):e71267. doi: 10.1002/cns.71267 (PMC13761291; doi:10.1002/cns.71267)
Supplement: Supplementary file 1 [file CNS-32-e71267-s001.docx]

Figure S1..Dose-response evaluation of STB treatment in SAMP8 mice .(A) Experimental design for evaluating the protective effects of different doses of STB in 2-month-old SAMP8 mice. Mice were randomly divided into four groups: control group, STB 10 mg/kg group, STB 60 mg/kg group, and STB 100 mg/kg group (n = 6 per group). STB was administered every other day for 3 months. (B) ABR thresholds at Click and 4, 8, 16, 24, and 32 kHz were measured before treatment and after 3 months of STB administration. Data are presented as mean ± SEM. Statistical analysis was performed using two-way repeated-measures ANOVA followed by appropriate multiple-comparison correction. p < 0.05.
